# Hypoxia Inducible Factor-1α Attenuates Ischemic Brain Damage by Modulating Inflammatory Response and Glial Activity

**DOI:** 10.3390/cells10061359

**Published:** 2021-06-01

**Authors:** Nashwa Amin, Shijia Chen, Qiannan Ren, Xiaoning Tan, Benson O. A. Botchway, Zhiying Hu, Fengpei Chen, Shan Ye, Xiaoxue Du, Zuobing Chen, Marong Fang

**Affiliations:** 1 Institute of Neuroscience, School of Medicine, Zhejiang University, Hangzhou 310058, China; 11818476@zju.edu.cn (N.A.); shijiachen@zju.edu.cn (S.C.); 616057635@zju.edu.cn (Q.R.); 21718583@zju.edu.cn (X.T.); benson009@zju.edu.cn (B.O.A.B.); 2 Department of Zoology, Faculty of Science, Aswan University, Aswan 81521, Egypt; 3 Obstetrics & Gynecology Department, Zhejiang Integrated Traditional and Western Medicine Hospital, Hangzhou 310003, China; huzhiying@zcmu.edu.cn; 4 The Second Clinical Medical College of Zhejiang Chinese Medical University, Hangzhou 310053, China; 201811112011282@zcmu.edu.cn (F.C.); 202011112611600@zcmu.edu.cn (S.Y.); 5 Translational Medicine Center, Affiliated Hangzhou First People’s Hospital, College of Medicine, Zhejiang University, Hangzhou 310000, China; dxiaoxue@zju.edu.cn; 6 Department of Rehabilitation Medicine, First Affiliated Hospital, College of Medicine, Zhejiang University, Hangzhou 310003, China

**Keywords:** endothelin-1, hypoxia-inducible factor-1, interleukin 10, astrocyte, dimethyloxalylglycine, acriflavine, proinflammatory cytokines

## Abstract

Hypoxia-inducible factor 1 can sufficiently control the progress of neurological symptoms after ischemic stroke owing to their actions associated with its downstream genes. In this study, we evaluated the role of HIF-1α in attenuating brain damage after endothelin-1 injection. Focal cerebral ischemia in mice were induced by endothelin-1 microinjection. Hypoxia-inducible factor 1 activator, dimethyloxalylglycine (DMOG), and HIF-1α inhibitor, acriflavine (ACF), were used to evaluate the hypoxia-inducible factor 1 activity during cerebral ischemia. The expression levels of HIF-1α, glial fibrillary acidic protein (GFAP), interleukin-10 (IL-10), inducible nitric oxide synthase (iNOS), phosphorylated I-kappa-B-alpha/total I-kappa-B-alpha (p-IκBα/IκBα) and nuclear factor kappa B (NF-kB) were assessed. Besides, mRNA levels of IL-10, tumor necrosis factor- alpha (TNF-α), and NF-kB were also analyzed. Results showed a noticeable increase in hypoxia-inducible factor 1 and IL-10 levels in the DMOG group with a decline in iNOS, TNF-α, and NF-kB levels, implying the anti-inflammatory role of hypoxia-inducible factor 1 activator following stroke. These findings were further corroborated by GFAP immunostaining that showed astrocytic activation to be inhibited 12 days post-ischemia, as well as histological and TEM analyses that demonstrated hypoxia-inducible factor 1 induction to alleviate neuronal soma damage and cell death. Based on our study, HIF-1α could be a potential therapeutic target for ischemic stroke.

## 1. Introduction

Cerebrovascular diseases, particularly ischemic stroke, have been widely studied because of their severe outcomes [1]. Considerable reduction of blood to defective tissue hinders oxygen (O_2_) and nutrient transportation, consequently causing cell death and tissue hypoxia [2]. To effectively deal with these harmful effects, the organ endeavors to augment oxygen supply to damaged tissues [3]. Induction of angiogenesis is an effective pathway towards increasing the oxygenation of hypoxic tissue. In this context, hypoxia-inducible factor 1 (HIF-1α) can regulate the expression of numerous genes, and consequently enhance the adaptability to minimal O_2_ environment [4]. It guides the genes related to angiogenesis, vasomotor regulation, and metabolism processes. Such genes can remarkably facilitate neuronal cells regeneration after ischemia-induced brain damage [5]. Previous studies reported that HIF-1α regulation and its accompanying impact on ischemic outcomes could be directed by the ischemic duration and degree of severity [6,7,8]. Also, previous reports revealed that the HIF-1α stabilization usually occurs by inhibiting the prolyl-4-hydroxylase domain (PHD) activity, which directs the upregulation of vascular endothelial growth factor (VEGF) and erythropoietin (Epo) expression, and alleviates the neurological deficit caused by ischemic stroke as well as edema [9,10,11].

The occurrence of cerebral ischemia releases a large number of cytokines, such as interleukin IL−1 and IL-6 from both glial cells and neurons in the injured part within a short period. These cytokines increase astroglia reactivity hyperplasia owing to astrocytes hypertrophy and proliferation [12]. Besides, the level of intermediate filament proteins can also be increased [13]. 

Astrocyte’s activity can significantly influence tissue survival after ischemia, such as water stability, blood brain barrier (BBB) regulation, glutamate homeostasis, blood flow, ionic balance, and elimination of neuroprotective factors [14,15]. Certain functions of astrocytes appear to play a considerable effect in contributing to the pathological outcomes after stroke. For instance, the participation of astrocytes in inflammatory response and their related neurotoxic substances may enhance stroke volume [16,17,18]. Also, astrocytes may release potentially harmful molecules, such as the proinflammatory cytokine (TNF-α), and reactive oxygen species (ROS) [19,20].

A previous study confirmed microinjection of endothelin-1 [ET-1] as an effective method to induce acute focal ischemia where striatum and parietal cortex were damaged [21,22,23]. This peptide is convenient for creating focal strokes that repeatedly takes place in the human brain [24,25,26]. Besides, establishing a stroke model by using ET-1 microinjection is easy to perform without any complicated surgical tactics of other stroke models, such as the MCAO technique [26,27,28].

In this study, the effectiveness of HIF-1α toward attenuating brain damage after ET-1 injection was evaluated. We found the inflammatory rate within the cortex of injured hemisphere to be reduced after HIF-1α activation. Moreover, the number of GFAP cells in white matter (WM), subventricular zone (SVZ), and striatum were decreased, implying that HIF-1α activation possesses a neuroprotective effect after ischemia by hindering astrocytes activation and pro-inflammatory cytokines. Histological and ultrastructure analyses of corresponding brain tissue after ischemia confirmed such pivotal role of HIF-1α activation. 

## 2. Materials and Methods

### 2.1. Animal Classification and Drug Administration

**Scheme 1:**
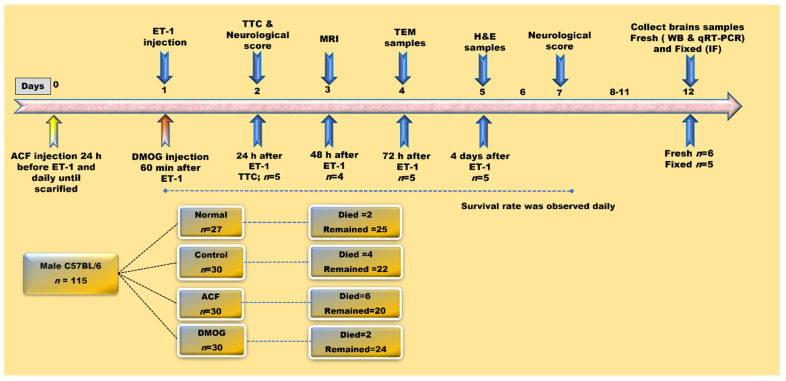
Representative experimental schematic course. ACF as HIF-1α inhibitor was injected 24 h prior to ET-1 injection, and daily for 12 days. DMOG as HIF-1α activator was injected once 60 min after ET-1 injection. 24 h post-ET-1 injection, TTC staining was employed to evaluate the infarct volume. 48 h after ET-1, MRI scan used to further confirm infract volume differences between groups. At days 4 and 5, 10 samples from each group were selected and sacrificed for TEM scan and H&E staining. On day 12, all animals were sacrificed, and brain samples were collected as fresh (directly kept in −80 °C for mRNA and protein extraction) and fixed samples (After perfusion, samples immersed in PFA 4% for 24 h prior to using them immunofluorescence studies). The neurological scores on day 1 after ET-1 injection, and day 7 were recorded in a blinded manner. *n* = number of animals per group, ET-1 = Endothelin-1, DMOG = Dimethyloxalylglycine, ACF = Acriflavine, Normal = without ET-1 injection, Control = ET-1 injection model without any treatment, HE = Hematoxylin and eosin staining, TTC = Triphenyl tetrazolium chloride, TEM = transmission electron microscope, MRI = Magnetic resonance image.

10-week-old 115 C57BL/6J male mice of weight 27 ± 2 g were randomly divided into four groups: Normal (*n* = 27) with no additional treatments; Control (*n* = 30). Mice were injected with a mixture of saline solution and DMSO 1% (Thermofisher, Netherlands, Cat: TS-20688) intraperitoneally (IP) at a dose of 0.2 mL/20 g body weight. This group represents mice with ET-1 administration; ACF (*n* = 30). Mice were treated with ACF (Sigma-Aldrich, China, Cat: A8251) dissolved in PBS with a concentration of 0.5 mmol/L and stored at 4 °C. The ACF solution was IP injected at a dose of 1.5 mg/kg body weight, 24 h before stroke and continually for 12 day up to sacrifice [29]. This family of mice reflects the HIF inhibitor group; DMOG (*n* = 30). Mice were administrated with DMOG (Selleck, China, Cat: S7483) dissolved in DMSO at a concentration of 5 mmol/L and whole DMOG/DMSO mixture was further diluted with a saline before being stored at −20 °C. Prior to injection, the obtained frozen solution was taken out and kept under atmospheric conditions for 10 min. The mice were IP injected with a one dose of 8 mg/kg, 60 min after ET-1 administration (Aladdin, China, Cat: E101621) [29]. Representative experimental schematic course is shown in the Scheme 1. 

### 2.2. Induction of Focal Cerebral Ischemia by Endothelin-1 

As an effective vasoconstrictor peptide, endothelin-1 (ET-1) was employed to induce focal cerebral ischemia. This was achieved by injecting ET-1 into the cranial area along the middle cerebral artery (MCA) by using a stereotaxic tool, producing a long-lasting vasoconstriction with a gradual reperfusion [30,31]. The injection site was identified with respect to bregma (1.0 mm anterior to the bregma, 1.2 mm lateral to the midline, and 1.6 mm below the pia). The time between inserting the needle and starting the ET-1 injection was 5 min, and the period between a full injection of ET-1 and syringe removal was also 5 min to minimize backflow. Each mouse received a dose of 2 μg ET-1 dissolved in 2 μL PBS (400 pmole/μL) by a 5 μL Hamilton syringe controlled by a syringe pump at a constant flow rate of 0.2 μL/min. 

### 2.3. Neurological Deficit Score and Survival Rate

The behavioral deficits and corresponding neurological scores were analyzed on days 1 and 7 following ET-1 injection [32]. The standards for different neurological scores were: 0 = no deficit observed; 1 = forelimb flexion; 2 = forelimb flexion combined with a lower lateral push resistance; 3 = unidirectional encirclement; and 4 = unidirectional encirclement integrated with a reduced conscious level. All behavioral analyses and statistics were conducted in a blinded mode. The animals were monitored daily up to day 12 after ET-1 injection for signs of moribundity and mortality. 

### 2.4. 2,3,5-. Triphenyl Tetrazolium Chloride [TTC] Staining 

After 24 h of ET-1 injection, mice were benumbed with isoflurane, their fresh brains carefully taken, and quickly frozen at −20 °C for 5 min. Coronal slices (1–2 mm) were created and dipped into 2% TTC solution (Sigma-Aldrich, St. Louis, MO, USA) for 20 min at 37 °C. The established sections were fixed overnight into 4% paraformaldehyde. The occurrence of infarction was identified by estimating the area negatively stained (white) with TTC. The existence of infarction in injured hemisphere was evaluated by probing the area negatively stained with TTC. The corresponding volume of damage was determined based on four slices with 2 mm specimen thickness and 3.7 to 6.3 mm relative to bregma. The volumes were calculated for each hemisphere, total brain size, ventricles, damaged tissue, and healthy tissue (remaining ipsilateral tissue). To evaluate the difference in hemisphere size resulting from edema and atrophy, the total ischemic damage was calculated according to the proportion of contralateral hemisphere using the following formula [7]:

$$\frac{\mathrm{contralateral}\;\mathrm{area}-\;\mathrm{remaining}\;\mathrm{ipsilateral}\;\mathrm{area}.}{\mathrm{contralateral}\;\mathrm{area}}$$

### 2.5. Hematoxylin-Eosin [HE] 

After perfusion and fixation, specimens were embedded into paraffin, and cut with 7 μm thickness. Briefly, de-paraffinization and re-hydration were carefully performed. The resulting sections were stained with hematoxylin solution for 6 min, dipped into acidic ethanol (1% HCl in 70% ethanol) for a few seconds, and rinsed in distilled water for 10 min. After, sections were stained with eosin solution for 5 min, followed by dehydration with a graded alcohol series before being cleared with xylene. Neutral balsam was used to seal the slides before further investigation by a light microscope instrument (Olympus BX61, Tokyo, Japan).

### 2.6. Western Blotting

The cortex region was dissected from brain samples and used to extract the protein. Cold RIPA buffer containing protease inhibitor cocktail was employed to grind the tissue using liquid nitrogen. After, the samples were collected and centrifuged at 12,000 rpm for 30 min at 4 °C. The supernatant was used to evaluate protein concentration by a BCA assay kit. All samples were adjusted to a concentration of 2 μg/μL. Prior to electrophoresis process, SDS loading buffer was mixed with samples and heated for 10 min in the boiling water for denaturation. The protein of ach sample (20 μg) was loaded to run an electrophoresis reaction in presence of SDS PAGE gel (10%) Fude Biological Technology, China at a constant voltage of 200 V for 50 min. Then, proteins were transferred to a polyvinylidene difluoride (PVDF) membrane with a constant current of 300 A for 70 min. The membranes were blocked with TBST containing skimmed milk (5%) for 3 h at room temperature and incubated with primary antibody at 4 °C overnight. The antibodies used were GAPDH ((CST, China, 1:1000), GFAP (CST, China, 1:1000), NF-kB (CST, China, 1:1000), and iNOS (Boster, China, 1:400) as well as HIF-1α (CST, China, 1:500)). Afterwards, membranes were washed with TBST containing 0.05% Tween 20 thrice, 5 min each. The membranes were incubated with secondary antibodies (goat anti-rabbit (Boster, China, 1:5000) and anti-mouse IgG antibody (Boster, China, 1:5000)) at room temperature for 2 h, and washed with TBST thrice, 5 min each. Finally, the membranes were exposed to hyper-film detection after incubation with the ECL system. The grayscale value of each band was analyzed using the Lab software. To ensure better reproducibility, each experiment was triplicated.

### 2.7. Immunofluorescence

After cardiac perfusion, brain tissues were collected, and kept in normal saline and paraformaldehyde (4%) overnight. After, tissues were transferred into a sucrose solution (30%) for three days. The samples were removed, directly embedded in OCT, immersed in liquid nitrogen, and then sliced into frozen sections with 18 um thickness by a frozen cryostat (Leica, Wetzlar, Germany). The resulting sections were dried at 37 °C, blocked with a blocking solution at room temperature for 1 h, and incubated with primary antibodies at 4 °C overnight under the following concentrations: anti-mouse GFAP (CST, 1:200) and anti-rabbit IL-10 (Abcam, China, 1:200). Next, samples were washed with 0.01 M (PBST) three times, 5 min each. After, sections were incubated with a secondary antibody (Alexa Fluor anti-mouse 488 for 3 h at room temperature) and rinsed with 0.01 M (PBS) three times, 5 min each. Finally, slides were coated with a mounting medium containing DAPI (VECTASHIELD, Burlingame, CA 94010, USA) and observed with a fluorescence microscope (Olympus BX51, Tokyo, Japan) at an excitation wavelength from 547 to 570 nm (Cy3, red), 494 to 520 nm (FITC, green), and 360 to 460 nm (DAPI, blue).

### 2.8. T2-Weighted MRI

A Biospec 3-T MRI system (Signa Excite HD, General Electric Medical System, Milwaukee, USA) was used to examine mice brains 2 days after ET-1 injection. Mice was anesthetized with pentobarbital sodium (0.3%), and intubated under mechanical ventilation at 60 beats/min. The temperature was adjusted to 37 °C, with heart and respiration rates tested. Precise lesion location was determined by the T2-weighted sequence and rapid-acquisition relaxation enhancement (time of repetition 5086 ms, echo time 70.1 ms with a resolution of 250_250_250 _m, and 15 slices).

### 2.9. Transmission Electron Microscopy

Perfusion of brain tissue was performed with normal saline solution and paraformaldehyde (4%). The brain was carefully removed, coronal slices (1 mm) were created, and submerged overnight in glutaraldehyde (2.5%) at 4 °C. Each sample was treated with potassium ferrocyanide (1%), reduced in osmium tetroxide (1%), dehydrated in graded acetones series, and embedded in Epon 812. Test sections were stained with lead citrate (0.25%) and uranyl acetate (5%) in methanol (50%) and inspected in a blinded manner. Such design enabled 10 representative neurons per each group as demonstrated by the corresponding nucleus and surrounding perikaryon. The ultrastructure was analyzed used a transmission electron microscope [Tecnai G2 Spirit 120 kV, New York, Thermo FEI, NY 1003, USA].

### 2.10. Real-Time PCR

The RNA expression levels of NF-kB, iNOS, TNF-α, and IL-10 in the cortex (Table 1) were detected by using quantitative Real-time PCR. β-actin was utilized as an internal control, and all primers were adopted by Primer Express software. The RNA in cortex was extracted based on the instructions on the Trizol RNA (Beyotime Biotechnology, China) extraction kit. RNA concentration was measured with a Nanodrop 2000 ultraviolet spectrophotometer (Thermo Fisher, Wilmington, DE 19,810 USA). According to the procedure of the DBI-2220 qPCR reverse transcription kit (Bestar, China) total RNA was reverse transcribed into cDNA, and quantitatively analyzed using Bestar SYBR Green qPCR master mix. The reaction parameters were: 50 °C for 2 min; 95 °C for 10 min; 40 cycles of 95 °C for 5 s, 55 °C for 30 s, and 72 °C for 30 s. The quality was probed by melt curve. The Bio-Rad CFX manager program (version 3.0.) was used to analyze the data, while the 2−∆∆Cq method was used to monitor the relative expression level of gene. To ensure a good reproducibility of data, each experiment was triplicated.

### 2.11. Statistical Analysis

Data were analyzed by one-way ANOVA using SPSS 20.0, followed by post-hoc Tukey test. A *p* value less than 0.05 was considered statistically significant. Histograms were generated in GraphPad Prism 5. All data are expressed as mean ± SEM. Gray values of Western blot results were calculated using Image Lab. Immunofluorescence and histological results were analyzed by Image-Pro Plus. * *p* < 0.05, ** *p* < 0.01, *** *p* < 0.001 was determined as statistically significant.

## 3. Results

### 3.1. HIF-1α Improved the Neurological Deficit Score

**Figure 1:**
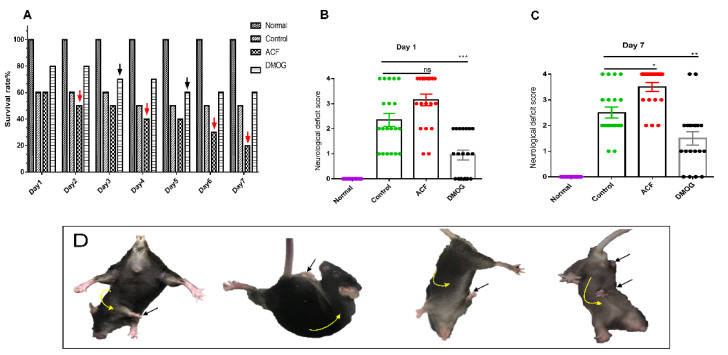
HIF-1α activation improves the neurological outcomes following ET-1 injection. (**A**) Survival rate %; DMOG group displays a higher survival rate, (**B**) Neurological deficit score Day 1: ACF vs. Control *p* = ns; DMOG vs. Control *** *p* < 0.001 (**C**) Neurological deficit score Day 7: ACF vs. Control * *p* < 0.05; DMOG vs. Control ** *p* < 0.01. (**D**) Represent the different observed neurological deficits. All values are expressed as mean ± SEM, One-way ANOVA with post hoc Tukey test, *n* = 20.

Figure 1 illustrates that the administration of DMOG following the ET-1 injection significantly improved the neurological deficit score compared with the control group on day 1 and day 7 (*p* < 0.001, Figure 1B, *p* < 0.01, Figure 1C, respectively). The ACF group showed a non-significant change compared with the control group on day 1 (Figure 1B) and a significant increase in the deficit score on day 7 (*p* < 0.05, Figure 1C). Observations of neurological deficit aspects are display in Figure 1D. Moreover, the survival rate in the DMOG group is higher than that of control and ACF groups as shown in Figure 1A. These findings imply that HIF-1α induction can attenuate the neurological deficit caused by cerebral ischemia.

### 3.2. HIF-1 Reduces the Infarct Area Volume Resulting from ET-1 Injection

**Figure 2:**
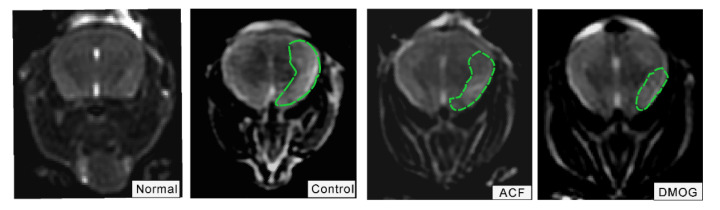
T2W Magnetic resonance image for mice brains following 48 h from ET-1 injection. The high-intensity region represents the infarct area [green dotted line]. DMOG group show smaller infarct volume than Control and ACF groups *n* = 4.

**Figure 3:**
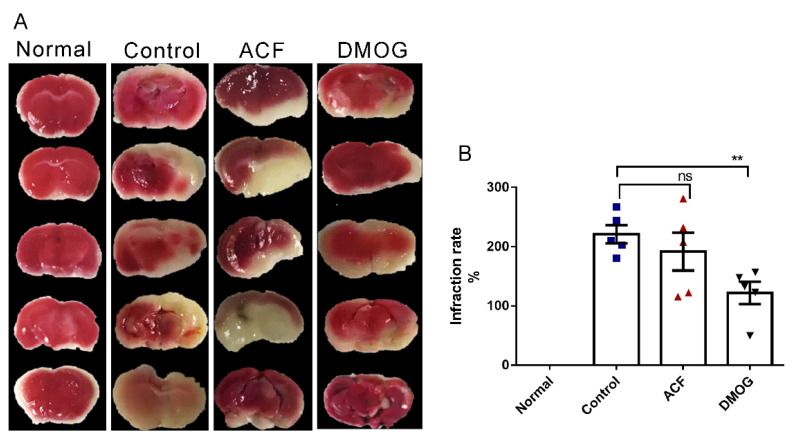
HIF-1α reduces the infarct volume following ET-1 injection. (**A**) TTC staining 24 h post ET-1 injection. (**B**) Infraction area rate, ACF vs. control *p* = ns, DMOG vs. Control: ** *p* < 0.01. All values are expressed as mean ± SEM, each group, one-way ANOVA, followed by post hoc Tukey test *n* = 5.

To examine the ischemia occurrence and variation of the injured area, the MRI technique and TTC staining were investigated and collected results are depicted in Figure 2 and Figure 3. The analysis of the TTC staining revealed a prominent reduction in the infarct area volume, consistent with the neurological results, and further emphasizes the role of HIF-1 in mitigating brain damage after ischemia. Where the infract volume of examined mice within the DMOG group showed a significant decrease compared with those in the Control group *p* < 0.01 (Figure 3B), but ACF mice were displayed a non-significant change compared with the control mice (Figure 3B).

### 3.3. HIF-1α Activation Recovered the Brain Damage Following ET-1 Injection

**Figure 4:**
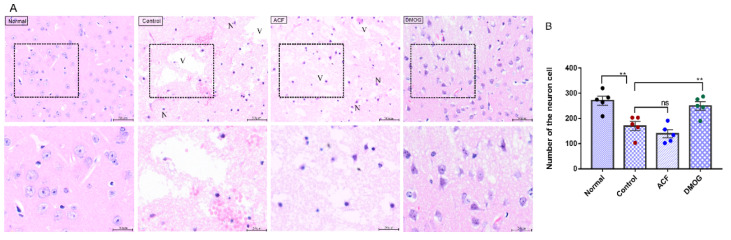
HIF-1α activation rescues neuron cells during cerebral ischemia. (**A**) H&E staining sections. Neuronal degeneration [N], vacuolated adjacent tissue [V]. (**B**) The number of neuron cells. Control vs. Normal: ** *p* < 0.01, ACF vs. Control: *p* = ns, DMOG vs. Control: ** *p* < 0.01. 50 μm & 20 μm. All values are expressed as mean ± SEM, one-way ANOVA, followed by post hoc Tukey test, *n* = 5.

Histological changes were observed in brain tissue by H&E staining after 4 days as shown in Figure 4.

The number of dead neurons was also calculated based on the darkly stained nuclei and cell body shrinkage [N] to inspect the existence of acute neuronal degeneration after ET-1. These changes were frequently associated with vacuolar alteration within the cytoplasm and soft vacuolated adjacent tissue [V]. The Control group showed a remarkable reduction in the number of surviving neuron cells after ischemia (*p* < 0.01) (Figure 4B), whereas an obvious increase of these cells has occurred in the case of the DMOG group (*p* < 0.01) (Figure 4B).

### 3.4. HIF-1α Activation Possesses a Neuroprotective Effect Post-Stroke

**Figure 5:**
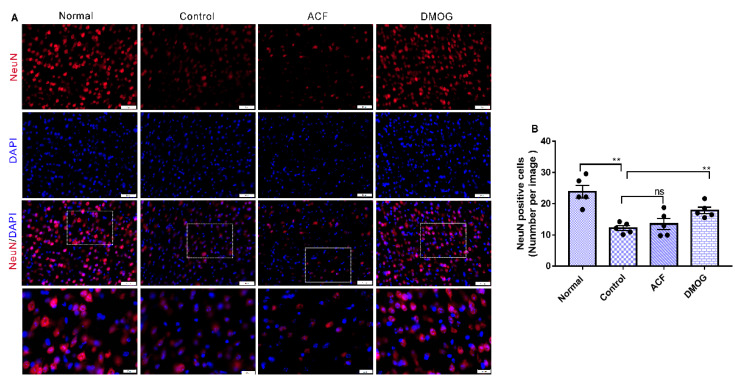
HIF-1 activation possesses a neuroprotective effect post-stroke. (**A**) NeuN immunostaining. (**B**) Number of NeuN positive cells post-stroke. Control vs. Normal; ** *p* < 0.01, ACF vs. Control; *p* = ns, DMOG vs. Control; ** *p* < 0.01. Scale bar 50 μm and 20 μm. All values are expressed as mean ± SEM, One-way ANOVA with post hoc Tukey test, *n* = 5.

To further validate the role of HIF-1 activation in rescuing neuronal cells after stroke, NeuN immunostaining was employed, and the results are presented in Figure 5A. The results showed a significant decrease of the NeuN positive cells number after ET-1 injection in the control group compared with normal group (*p* < 0.01). However, the administration of HIF-1 activator displayed a protective effect and significantly increased the NeuN positive cells (*p* < 0.01) compared with control group. Meanwhile, the administration of HIF-1 inhibitor resulted in a non-significant improvement in the neuron rescue compared with control group following ET-1 injection (Figure 5B). These outcomes are consistent with the HE staining results, demonstrating that HIF-1 activation can sufficiently alleviate the neuron damage after ischemia.

### 3.5. HIF-1α Activation Can Modulate Brain Damage via Neuronal Soma Protection

**Figure 6:**
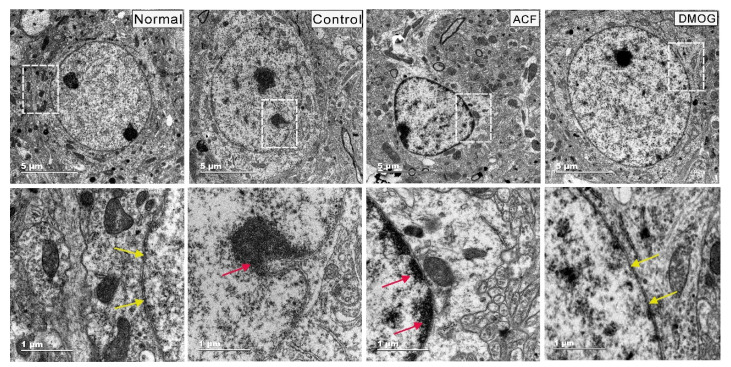
HIF-1α activation alleviates brain damage via protecting the neuronal soma. Representative images of transmission electron microscopy [TEM] [magnification ×24,000]. The ultrastructure of the neuronal soma 7 days post-ischemia, yellow arrow points to the nuclear membrane which shows no alteration in case of DMOG, ACF and Control groups show a significant alteration in the neuronal soma structure when compared with the normal mice. Scale bar: 5 μm and 1 μm, *n* = 5.

As an important marker for brain damage, neuronal soma microstructure after ischemia was characterized by a TEM scan. Brain tissue adopted from normal animals displayed healthy neuronal soma demonstrated by its rounded shape. As shown in Figure 6, the nuclear envelope is intact with several distinct nucleoli, and the DNA illustrates normal compaction. Neuronal somas of Control and ACF groups exhibited a loss of nuclear envelope or cells with irregularly shaped nuclei. Moreover, numerous nuclear indentations, more bundles of nuclear filaments, and chromatin clumps against the double nuclear envelope have also existed. On the other hand, the DMOG group showed significant enhancements in the neuronal soma structure close to the normal shape.

### 3.6. HIF-1α Reduces the Glial Activity Following ET-1 Injection

**Figure 7:**
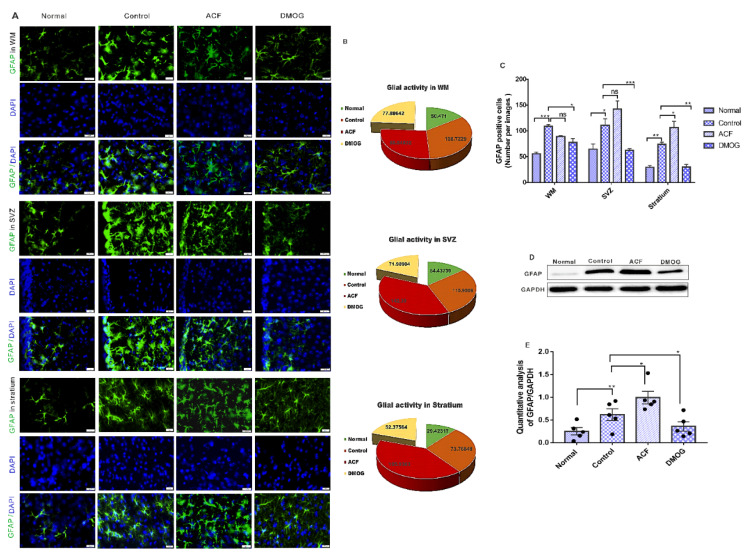
HIF-1α reduces the glial activation after ET-1 injection in WM, SVZ and striatum. (**A**) Immunofluorescence staining of GFAP positive cell in WM, SVZ and Striatum, Scale bar 20 μm, (**B**) 3D-Pie diagram show the proportion of whole GFAP positive cells within each area (**C**) Number of GFAP positive cells; Control Vs. Normal WM *** *p* < 0.001; SVZ * *p* < 0.05; Striatum * *p* < 0.01, DMOG Vs. Control WM * *p* < 0.05; SVZ *** *p* < 0.001; Striatum ** *p* < 0.01, ACF Vs. Control WM *p* = ns; SVZ *p* = ns; Striatum * *p* < 0.05, all values are expressed as mean ± SEM, one- way ANOVA, followed by post hoc Tukey test, *n* = 5. (**D**) Western blot, (**E**) Quantitative analysis of GFAP, Control vs. Normal ** *p* < 0.01, DMOG vs. Control * *p* < 0.05, ACF vs. Control * *p* < 0.05. all values are expressed as mean ± SEM, one- way ANOVA, followed by post hoc Tukey test, *n* = 5.

To confirm the effect of HIF-1α on neuroinflammation, the potential reactive astrogliosis in the WM, striatum, and SVZ areas after 12 days of reperfusion were measured by immunofluorescence analysis of GFAP-positive astrocytes as illustrated in Figure 7A. The DMOG group displayed a noticeable decrease in the WM, SVZ, and striatum parts (*p* < 0.01, *p* < 0.01, and *p* < 0.001, respectively) (Figure 7B,C) when compared with the Control group. In contrast, the ACF group presented a significant increase in the number of GFAP-positive cells in both SVZ, and striatum zones compared with the control group (*p* < 0.01, *p* < 0.01, respectively). The Western blot analysis of the DMOG group bands exhibited a significant reduction in GFAP expression in comparison with the control mice (*p* < 0.05) (Figure 7D,E). In contrast, control group compared with normal group displayed a significant elevation in GFAP expression (*p* < 0.01) (Figure 7D,E), as well as ACF group showed a significant increase in GFAP protein expression (*p* < 0.05) (Figure 7D,E). These results reflect the inhibitory effects of HIF-1α on GFAP-positive astrocytes and thereby conform its contribution to the neuroprotection effect.

### 3.7. HIF-1α Activation Down-Regulates the Pro-Inflammatory Cytokines iNOS, NF-kB, and Upregulates the Anti-Inflammatory Marker IL-10

**Figure 8:**
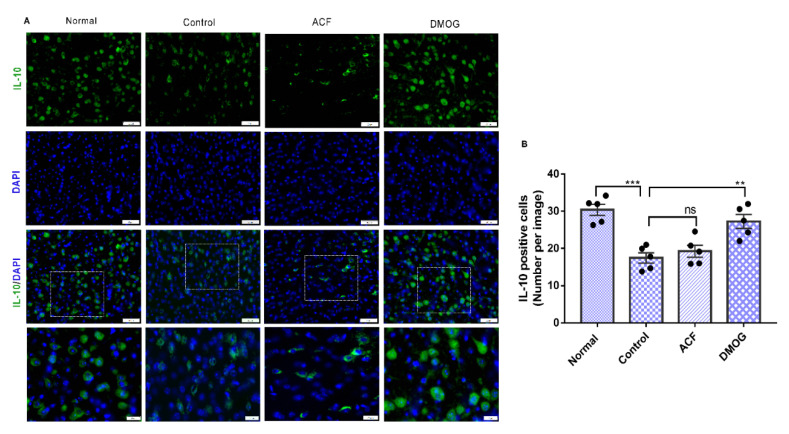
HIF-1α activation attenuates inflammation via inducing the anti-inflammatory cytokine IL-10. (**A**) Immunofluorescence staining of IL-10, Scale bar 50 μm and 20 μm (**B**) Number of IL-10 positive cells. Control vs. Normal *** *p* < 0.001; ACF vs. Control *p* = ns; DMOG vs. Control ** *p* < 0.01. All values are expressed as mean ± SEM, one- way ANOVA, followed by post hoc Tukey test, *n* = 5.

**Figure 9:**
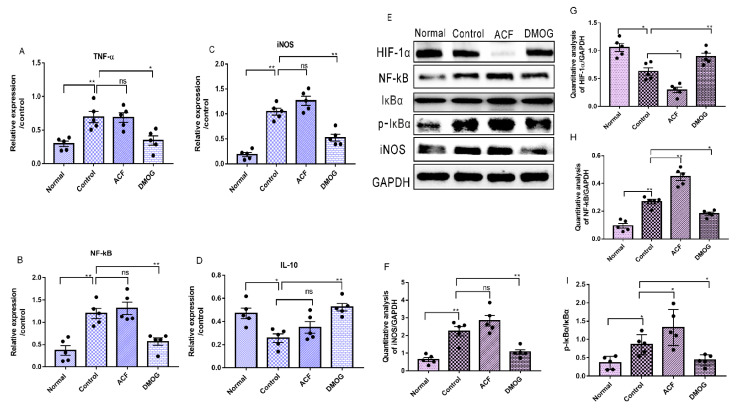
HIF-1α activation alleviates brain damage via decreasing the expression level of pro-inflammatory cytokines. (**A**) mRNA level of TNF-ἀ; Control Vs. Normal ** *p* < 0.01, ACF vs. control * *p* < 0.05, DMOG vs. Control * *p* < 0.05, (**B**) mRNA level of NF-kB; Control Vs. Normal ** *p* < 0.01, ACF vs. control *p* = ns, DMOG vs. Control ** *p* < 0. 01, (**C**) mRNA level of iNOS; Control Vs. Normal ** *p* < 0.01, ACF vs. control *p* = ns, DMOG vs. Control ** *p* < 0.01 and (**D**) mRNA level of IL-10; Control Vs. Normal ** *p* < 0.05, ACF vs. control *p* = ns, DMOG vs. Control ** *p* < 0.01. (**E**) Representative western Blot, (**G**) Quantification analysis of HIF-1α; Control vs. Normal * *p* < 0.05 ACF vs. Control *p* = ns, DMOG vs. Control ** *p* < 0.01, (**F**) Quantification analysis of iNOS; Control vs. Normal ** *p* < 0.01, ACF vs. Control *p* = ns, DMOG vs. Control ** *p* < 0.01, (**H**) Quantification analysis of NF-kB; Control Vs. Normal ** *p* < 0.01, ACF vs. Control * *p* < 0.01, DMOG vs. Control * *p* < 0.05. (**I**) Normalize analysis of p-IκBα/IκBα; Control vs. Normal * *p* < 0.05, ACF vs. Control * *p* < 0.05, DMOG vs. Control * *p* < 0.05. All values are expressed as mean ± SEM, one-way ANOVA, followed by post hoc Tukey test, *n* = 5.

Inflammation represents the first-line response to brain damage following cerebral ischemia. To test the effectiveness of HIF-1α activation 12 days post-ischemia upon the inflammatory response, Western blot, immunofluorescence, and RT-PCR were performed to detect the expression level of inflammatory cytokines IL-10, NF-kB, p-IκBα/IκBα and iNOS as well as HIF-1α. The immunofluorescence staining analysis of IL-10 positive cells and its mRNA level for the DMOG group (Figure 8A,B) (Figure 9D) revealed a significant enhancement (*p* < 0.01 and *p* < 0.01, respectively) compared with those of the control group.

The HIF-1α protein expression of both control and ACF groups displayed a lower level when compared with the normal group. However, the DMOG group showed a higher level of HIF-1α expression 12 days post-ischemia (Figure 9G) (*p* < 0.001). Besides, the control group showed high expression levels of iNOS, NF-kB and p-IκBα/IκBα after ET-1 injection when compared with the normal group (Figure 9F,H,I) (*p* < 0.01, *p* < 0.01 and *p* < 0.05) respectively. A similar course was noted in case of the ACF group compared with the control group as shown in Figure 9F,H,I (*p* =ns, *p* < 0.01 and *p* < 0.05) respectively.

In contrast, the DMOG group displayed a lower mRNA level of iNOS, NF-kB, and TNF-ἀ (*p* < 0.01, *p* < 0.01, and *p* < 0.05, respectively) than the control group together with a similar trend of iNOS and NF-kB protein expression as well as p-IκBα/IκBα level (*p* < 0.01, *p* < 0.05, and *p* < 0.05 respectively) (Figure 9A–C,F,H,I). This indicates the importance of HIF-1α activation as an efficient strategy to mitigate the inflammatory response following cerebral ischemia.

## 4. Discussion

This study explained the neuroprotective effect of HIF-1α 12 days post-microinjection of ET-1 into the brain of adult mice. The neuroprotective effect of HIF-1α was mediated by modulating neuroinflammation in the ischemic brain and glial activity in the WM, SVZ, and striatum regions. HIF-1α activation 12 days post-stroke ameliorated neurological deficit and improved the survival rate. Besides, HIF-1α induction reduced the infarct volume 24 h and 48 h post-stroke. DMOG, as an HIF-1α activator, possesses an inhibitory effect on pro-inflammatory cytokines iNOS, NF-κB, and TNF-α.

In the present study, we found DMOG to upregulate the anti-inflammatory cytokine, IL-10, in the brain tissue. Furthermore, HIF-1α activation can modulate glial activity 12 days post-stroke. Microstructure observations of neuronal soma at 72 h post-stroke showed that HIF-1α activation can improve damaged brain tissue. These results were consistent with histological outcomes, which showed a significant increase in neuronal cell number following HIF-1α induction after stroke. Therefore, HIF-1α may protect brain tissue post-stroke by modifying inflammatory and glial responses in brain tissue 12 days post-stroke. Under normal oxygen levels, HIF-1α protein can be degraded by the PHD enzyme within a short time. In hypoxic conditions, however, HIF-1α protein is stabilized and accumulated [3,33]. HIF-1α moves to the nucleus and establishes a heterodimer with the β-subunit of the aryl hydrocarbon receptor nuclear translocator (ARNT) after being stabilized. Afterwards, it binds to hypoxia-response elements, and promotes the expression of more than 60 genes [29,34].

Oxidative stress considerably affects the progress of pathological features post-stroke [35]. A recent showed HIF-1α to be very sensitive to oxidative attacks, which may promote the degradation process of HIF-1α [36]. Besides, HIF-1α may transcriptionally induce its degradation under hypoxia [37,38]. The modulation of HIF-1α activity can occur by using PHD inhibitors and drugs that interfere with heterodimer formation [29]. In this study, we employed the PHD inhibitor, DMOG, to activate HIF-1α, while the heterodimer inhibitor, ACF, was used to inhibit HIF-1α activity. Previous studies have reported HIF-1α activation to alleviate brain injury induced by stroke [29,30,31,32,33,34]. Moreover, HIF-1α is an inflammation mediator, as it is stabilized by IL-1b and TNF-α and is paramount to leukocyte survival [30]. In ascertaining the contribution of HIF-1α to stroke recovery, we found that DMOG enhanced neuronal cell survival, and improved neuronal soma deficits that had been induced by stroke. To examine whether the protective effect of HIF-1α after stroke is achieved by modulating inflammation, we evaluated the expression levels and mRNA of pro-inflammatory cytokines (iNOS, TNF-α, NF-κB and p-IκBα/IκBα), and anti-inflammatory cytokines (IL-10) in the ischemic brain. DMOG upregulated IL-10 protein expression and mRNA levels 7 days post-stroke. On the contrary, ACF showed a downregulated IL-10 level in the ischemic brain. Our results suggest that HIF-1α improves stroke outcomes by enhancing IL-10 levels, and curtailing iNOS, TNF-α and NF-kB levels. Inflammatory reaction has detrimental effects after ischemia. Therefore, the inhibition of inflammatory response could be a therapeutic strategy [28]. Also, our results showed a lower number of GFAP-positive cells in the injured hemisphere, indicating that HIF-1α may inhibit astrocytes and microglia caused by the injury.

Activation of microglia is a major occurrence in ischemic stroke and is significantly involved in the pathological progression and neuroinflammation response of ischemic tissue. This activation of microglia comprises multiple stereotypical aspects, such as polarization, proliferation, and morphological changes [39,40]. Acute ischemia causes a complete transformation of microglia into amoeboid shapes and de-ramification. Microglial activation during ischemia may change according to the affected area. Moreover, the complexity of microglia branching after focal cerebral ischemia increases within the striatal region and decreases in cortical regions. Hence, microglia can act as either de-ramified or hyper-ramified in response to focal ischemia, with this action dependent on the degree of ischemia or brain regions. According to ischemic conditions, cells usually adapt throughout motivating the HIF-1 [41,42]. The role of HIF-1 varies largely based on the different types of cells, elucidating the effective role of microglia as an essential neuroinflammation mediator in various brain diseases [43,44].

In summary, we have provided strong evidence on the effectiveness of HIF-1α activation in alleviating brain damage after ischemic stroke. This was due to its ability to modulate neuroinflammation and glial activity. Moreover, HIF-1α activation can significantly control astrocyte reactivity at WM, SVZ, and striatum zones within the injured hemisphere by inhibiting pro-inflammatory cytokines. The above findings were corroborated by the minimization of neuronal cell death, as NeuN positive cells were considerably increased after HIF-1α induction. This study may open new avenues for the development of an effective treatment that mitigates inflammation of brain damage after stroke.

## Figures and Tables

**Table 1:** Primer sequences for RT-qPCR which used were as follows.

Gene	Forward Primer	Reverse Primer
β-actin	5′-CTGTCCCTGTATGCCTCTG-3′.	5′-ATGTCACGCACGATTTCC-3′.
IL-10	5′-CGGGAAGACAATAACTGCACCC-3′	5′-CGGTTAGCAGTATGTTGTCCAGC-3′
NF-kB	5′-GCTGCCAAAGAAGGACACGACA-3′	5′-GGCAGGCTATTGCTCATCACAG-3′
iNOS	5′-CACCTTGGAGTTCACCCAGT-3′	5′-ACCACTCGTACTTGGGATGC-3′
TNF-ἀ	5′-GGTGCCTATGTCTCAGCCTCTT-3′	5′-GCCATAGAACTGATGAGAGGGAG-3′

## Data Availability

The datasets during and/or analyzed during the current study are available from the corresponding author on reasonable request.
